# Reviewing the blood ordering schedule for elective orthopedic surgeries at a level one trauma care center

**DOI:** 10.4103/0974-2700.66521

**Published:** 2010

**Authors:** Arulselvi Subramanian, Kanchana Rangarajan, Sudeep Kumar, Vijay Sharma, Kamran Farooque, Mahesh Chandra Misra

**Affiliations:** Blood Bank & Department of Laboratory Medicine, Jai Prakash Narayan Apex Trauma Centre, All India Institute of Medical Sciences, AIIMS, New Delhi, India; 1 Department of Orthopaedics, Jai Prakash Narayan Apex Trauma Centre, All India Institute of Medical Sciences, AIIMS, New Delhi, India

**Keywords:** Audit, blood ordering schedule, elective orthopedic surgeries, surgical blood ordering equation, transfusion

## Abstract

**Background:**

Patients undergoing elective orthopedic surgeries often incur excess blood loss necessitating transfusion. The preoperative placement of blood requests frequently overshoots the actual need resulting in unnecessary crossmatching.

**Aims:**

Our primary goal was to audit the blood utilization in elective orthopedic surgeries in our hospital over a 1-year period and recommend a blood ordering schedule.

**Materials and Methods:**

A retrospective analysis of patients who underwent elective orthopedic surgeries over a period of 1 year was done. The data collected include patients’ age, sex, type of surgical procedure, pre- and postoperative hemoglobin (Hb) levels, number of units crossmatched, returned, transfused, crossmatch to transfusion ratio (C:T), transfusion indices, estimated blood loss for each surgical procedure, and the actual and predicted fall in Hb. We propose a blood ordering schedule based on surgical blood ordering equation.

**Results and Conclusions:**

A total of 487 patients with a median age of 37±17 years (mean ± standard deviation) were evaluated. One thousand three hundred and seventy-seven units of blood were crossmatched and only 564 units were transfused to 260 patients. Fifty-nine percent of the units crossmatched were not transfused. Six of the 12 elective procedures had a C:T ratio higher than 2.5. Ten of the 12 procedures (83.3%) had a low transfusion index (TI < 0.5). The calculated red blood cell units were less than 0.5 in 5 of the 12 elective procedures, and hence we recommend a group and save policy for these procedures. Blood ordering schedule based on patient and surgical variables would provide an efficient way of blood utilization and management of resources.

## INTRODUCTION

Many elective orthopedic surgeries most often, inevitably lead to excess blood loss during the procedure, requiring transfusion. The preoperative assessment of blood requirements is often an overassumption as shown by blood bank registers. The consequences of such misuse include outdating of blood, overburdening of blood bank personnel, depletion of blood bank resources, and wastage of time.[1–3] When a blood bag is taken out of blood inventory for preoperative crossmatch, it becomes unavailable for other patients’ use. Many a time the newer blood bags get issued earlier than the older bags, which are kept matched and reserved for elective surgeries. Hence it is quite necessary to streamline the blood usage by incorporating a blood ordering schedule for such procedures.

The maximum surgical blood ordering schedule (MSBOS) is a list of common elective surgical procedures for which the maximum number of units of blood are crossmatched preoperatively for each procedure.[4,5] It is basically designed to order enough blood for 85%–90% of patients for each surgical procedure. The ratio of the number of units crossmatched to the number of units actually transfused, that is, C:T ratio should not exceed 2:1.[6] Although MSBOS has improved the efficiency of blood utilization, there are certain drawbacks, the most significant one being the absence of accountability for individual differences in transfusion requirements between different persons undergoing the same surgical procedure.[7]

Surgical blood ordering equation (SBOE) is an extended MSBOS incorporating patient and surgical variables, such as pre- and postoperative hemoglobin (Hb) levels of the patient and the amount of surgical blood loss during each surgical procedure.[8,9] By establishing such an SBOE, each surgical team can develop its own transfusion system and set its own transfusion limits. They can also audit the operative blood loss for each procedure.[10]

The main aim of this present study is to improve the efficiency of blood utilization in trauma care blood bank and reduce unnecessary crossmatching as well as wastage of blood bank resources. Our primary objective is to audit the blood utilization in elective orthopedic surgeries and recommend a SBOS based on SBOE.

## MATERIALS AND METHODS

Case records of patients who underwent elective orthopedic surgeries and their respective blood bank records were collected. A retrospective analysis of data for a period of 1 year from January to December 2007 was done. Patients who underwent massive transfusion were excluded from the study to eliminate bias. Massive transfusion in a trauma setting has been defined in several ways. Definitions based on the absolute number of transfused red blood cells (RBCs) stipulate either the transfusion of 10 units or more of packed RBCs in 24 h or the transfusion of 20 units of RBC or more in the course of the hospital stay.[11] Our definition of massive transfusion includes those patients who received 10 units or more of packed RBCs in 24 h. Patient and surgical parameters include age and gender, type of surgical procedure, pre- and postoperative Hb levels, estimated blood loss for each surgical procedure, and predicted fall in Hb. Routine transfusion parameters, such as the number of units transfused, crossmatched, and returned, were tabulated. The calculated indices include crossmatch to transfusion ratio (C:T ratio), transfusion probability (%T), and transfusion index (TI).

C: T ratio = No of units cross matched ÷No of units Transfused

Transfusion probability (%T) = (No of patients Transfused ÷ No of patients cross matched) × 100

Transfusion Index (TI) = No of units Transfused ÷ No of units cross matched

A realistic objective for C:T ratio is 1–2:1. A C:T ratio >2.5, %T > 50, TI > 0.5 is considered indicative of significant blood utilization.[3,4,6,12] We calculated a blood ordering schedule using the SBOE. Many such SBOEs are in use; however, we used the SBOE proposed by Nuttall *et al*.[10] for purposes of simplicity,

Number of RBC units required = predicted Hb fall (g/dL) – [preoperative Hb (g/dL) – postoperative threshold Hb (g/dL)]

The predicted Hb fall is calculated based on the amount of blood loss during each surgical procedure assuming that 1 unit of blood lost will decrease the patient’s Hb by 1 g/dL. The postoperative Hb is taken at 24 h postsurgery. The difference in the mean preoperative and mean postoperative Hb levels of the patient for each procedure gives the actual Hb losses for any surgical procedure.

## RESULTS

A total of 487 patients were included in our study. These patients underwent 12 different elective procedures in orthopedic surgery in our trauma center. There were 390 males (80%) and 97 females (20%). The mean age was 37±17 years (mean ± standard deviation [SD]). Out of the total 1377 RBC units crossmatched, only 564 units (41%) were transfused to 260 patients. Fifty-nine percent of the total crossmatched units were not transfused. The number of patients and units, cross-matched and transfused is tabulated in Table 1.

**Table 1:** Blood crossmatch and transfusion patterns for different elective orthopedic surgeries

Type of surgery	Crossmatched		Transfused	
	Patients (n)	Units (n)	Patients (n)	Units (n)
Internal fixation with compression plating	165	444	72	147
Interlocking nail	181	505	109	246
Dynamic compression screw	21	66	11	23
Dynamic hip screw	26	65	11	16
External fixation	17	52	11	29
Hip prosthetic replacement	21	75	9	18
Decompression with fixation for # spine	27	83	18	35
K-wire fixation	5	6	3	4
Tension-band wiring	4	14	2	6
Enders nail for femoral #	13	37	9	19
Amputations	5	27	4	20
Lumbar discectomy	2	3	1	1

# FRACTURE

A majority of the patients (71%) admitted with trauma underwent internal fixation with compression plating (*n* = 165) and interlocking nailing (*n* = 181). More than half of the units crossmatched for these 2 surgeries (556/949 units) were not transfused to these patients. The C:T ratio, transfusion probability, as well as transfusion index were formulated for each of the elective procedures and is shown in Table 2.

Six of the 12 elective procedures had a high C:T ratio > 2.5 with 2 of them, namely, dynamic hip screw and hip prosthetic replacement exceeding 4. However, the probability that a patient would undergo transfusion (%T) was <50 in these procedures. All the surgeries except decompression with fixation for fracture spine and tension-band wiring had 2 of the 3 indices raised indicating significant blood utilization in most (11/12) of the procedures. None of these surgeries had all the3 indices elevated.

**Table 2:** Blood utilization for different elective orthopedic procedures

Type of surgery	C:T ratio	%T	TI
Internal fixation with compression plating	3.02	43.64	0.33
Interlocking nail	2.05	60.22	0.5
Dynamic compression screw	2.87	52.38	0.35
Dynamic hip screw	4.06	42.3	0.25
External fixation	1.79	64.7	0.56
Hip prosthetic replacement	4.17	42.86	0.24
Decompression with fixation for # spine	2.37	66.67	0.42
K-wire fixation	1.5	60	0.67
Tension-band wiring	2.33	50	0.43
Enders nail for femoral #	1.95	69.23	0.5
Amputations	1.35	80	0.74
Lumbar discectomy	3	50	0.33

C:T RATIO: CROSSMATCH TO TRANSFUSION RATIO; %T: TRANSFUSION PROBABILITY; TI: TRANSFUSION INDEX.

# FRACTURE

The minimum, average, and maximum blood loss for each surgical procedure was estimated by the Orthopedicians based on mop counts and the amount of suction fluid [Table 3]. This excludes the amount of fluid infused at the time of surgery. The corresponding Hb loss was predicted based on the assumption that 1 unit of blood loss decreases the Hb by 1 g/dL. The mean preoperative Hb in the study group was 10.51±0.8 g/dL (mean ± SD) and mean postoperative Hb was 10.17±0.9 g/dL (mean ± SD). The reduction in Hb in the present study is the result of the reduction in Hb prior to and after operation in 487 subjects under study. Their preoperative Hb was stabilized with blood transfusions prior to the planned procedure if it was low. On the other hand, blood ordered preoperatively before the elective procedures was in excess of the actual need as shown by the blood loss during each surgical procedure [Table 3]. Similarly, the postoperative transfusions were given 24 h after surgery when required

**Table 3:** Blood loss during the surgery

Type of surgery	Minimum (mL)	Average (mL)	Maximum (mL)
Internal fixation with compression plating	50	75–100	200
Interlocking nail	30–50	75	100
Dynamic compression screw	150	250	350
Dynamic hip screw	100	200	300
External fixation	50	75	100
Hip prosthetic replacement	100	200	300
Decompression with fixation for # spine	200	300–350	400–500
K-wire fixation	100	150	200
Tension-band wiring	50	100	150–200
Enders nail for femoral #	20	50	100
Amputations	100	150	300
Lumbar discectomy	50	100	150

# FRACTURE

**Figure 1:**
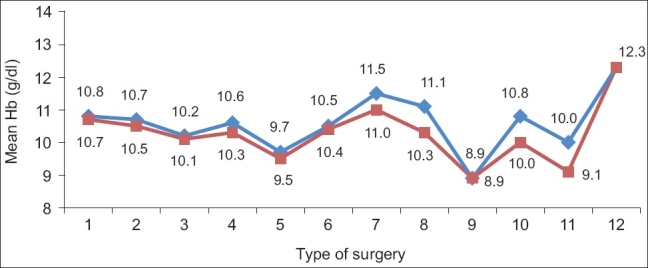
Actual Hb fall (g/dL). 1: Internal fixation with compression plating, 2: interlocking nail, 3: dynamic compression screw, 4: dynamic hip screw, 5: external fixation, 6: hip prosthetic replacement, 7: decompression with fixation for # spine, 8: K-wire fixation, 9: tension-band wiring, 10: Ender nail for femoral #, 11: amputations, 12: lumbar discectomy

The actual Hb fall [Figure 1] was much lower in most of the procedures than that predicted from the amount of surgical blood loss. The SBOS was drafted based on the SBOE. When the number of units calculated is less than 0.5 units, a group and save policy is recommended. When it is more than 0.5 units, the number of RBC units is rounded off to the nearest integer. The actual, predicted Hb loss, and the SBOS is tabulated in Table 4.

**Table 4:** Hemoglobin loss–actual and predicted with the surgical blood ordering schedule

Type of surgery	Predicted Hb loss (g/dL)	Actual Hb loss (g/dL)	No of red blood cells required (units)	Surgical blood ordering schedule (units)
Internal fixation with compression plating	0.8	0.08	0.7	1
Interlocking nail	0.4	0.20	0.2	G and S
Dynamic compression screw	1.4	0.09	1.3	1
Dynamic hip screw	1.2	0.31	0.9	1
External fixation	0.4	0.2	0.2	G and S
Hip prosthetic replacement	1.2	0.17	1.03	1
Decompression with fixation for # spine	1.6–2.0	0.50	1.5	2
K-wire fixation	0.8	0.8	0	G and S
Tension-band wiring	0.6–0.8	0.05	0.6–0.8	1
Enders nail for femoral #	0.4	0.81	0.4	G and S
Amputations	1.2	0.3	0.9	1
Lumbar discectomy	0.6	0	0.6	1

HB: HEMOGLOBIN

# FRACTURE

## DISCUSSION

Blood is a precious commodity and its proper utilization is the key for efficient management of blood bank resources. Blood and blood components are critical in patient care but are in limited supply and carry numerous risks and significant cost. A careful assessment of the risks and benefits of allogenic transfusion is essential for a good patient outcome. The risk of transfusion transmitted infections, such as human immunodeficiency virus (HIV) and hepatitis B and C, as well as emerging infections, such as the new variant Creutzfeldt–Jacob disease, pose a significant threat to those patients needing transfusion. The estimated risks of transfusion have dramatically decreased over the recent years as increased test sensitivity has reduced infectious window periods.[13] Advanced blood screening techniques, such as the nucleic acid test, have been introduced to improve the quality of blood products. Hence it is essential that the usage of blood and blood products be rationalized and they are saved for crisis situations.

Appropriate placement of blood requests according to a planned schedule most often averts the consequences of indiscriminate ordering of blood. This requires streamlining blood ordering schedule keeping in view the blood bank resources, time, as well as money. MSBOS has been in use since 1975[4] and has been undergoing periodic modifications since the time of implementation. The initial formulation of MSBOS was done using Mead’s criterion.[1–3,12] According to this criterion, the number of RBCs calculated was one and half times the transfusion index for each surgical procedure. Bhutia *et al*.[1] evaluated the preoperative blood ordering and transfusion practices for common elective general surgical procedures using the Mead’s criterion. In this study, 40% of the crossmatches performed were found to be unnecessary. A maximum SBOS was drafted based on these data. In a similar study by Vibhute *et al*,[3] the blood evaluation and transfusion practices for 500 elective general surgical procedures were evaluated and MSBOS was put into immediate effect after formulation. As a result, the blood ordering pattern changed for the next 150 patients. This shows that MSBOS definitely improves the blood utilization and reduces the wastage rate. However, it does not take into consideration the individual differences in transfusion needs between different patients undergoing the same surgery.

SBOEs are an evolved form of MSBOS incorporating patient and surgical variables. Many risk factors have been analyzed and found to be useful in predicting the blood transfusion.[8,9] Some of them include low preoperative Hb/hematocrit, short stature, female sex, availability of preoperative autologous blood donation, surgical blood loss, and the type of surgery. In the present study, none of our patients had preoperative autologous blood available. We recommend a draft, SBOS formulated using the SBOE proposed by Nuttall *et al*.[10] In this, the number of RBC units required is calculated incorporating patient-specific factors, such as the mean pre- and postoperative Hb levels of the patient and the amount of surgical blood loss predicted by the surgeons. The amount of blood loss was predicted based on the surgical blood loss during each procedure. This would provide an easier way of finding the required amount of RBCs to be cross matched preoperatively.

SBOEs were used in many studies.[10,14,15] In a retrospective review[14] of 182 cases of spinal instrumentation and fusion surgery, incorporating patient factors resulted in increased efficiency of blood ordering practices as evidenced by a lower C:T ratio with the SBOE than with the MSBOS system. In another retrospective study[15] comparing the SBOE with the MSBOS, SBOE reduced the C:T ratio from 1.2 to 1.6 but reduced the ordered units in statistical significance in only 2 of 6 types of surgeries. Prospective studies using this SBOE would be necessary to study the efficiency of the blood ordering system in a trauma setup.

The concept of “group and save” has been in use since many years and many authors have shown that merits outweigh the perceived demerits.[16,17] In the present study, when the number of units calculated for any surgery is less than 0.5 units, a group and save policy would be ideal. In this group and save policy, no presurgical crossmatch would be done; instead grouping followed by screening for antibody is performed. In this study, group and save policy was recommended in 5 of the 12 elective procedures. Also none of the elective procedures required more than 2 units of packed cells.

Type and screen is suggested to be 99% effective in preventing incompatible transfusions.[18] This is due to the high efficacy of antibody screening in the detection of potentially clinically significant antibodies. According to the American Association of Blood Banks recommendations,[19] if the antibody screening is negative and there are no previous records of detecting such antibodies, a serological testing to detect ABO incompatibility is adequate and antiglobulin testing in performing crossmatch may be skipped. Benefits of such a type and screen (T and S) include reduced cost of reagents (used for crossmatch), improved turnaround time, and decreased workload of the laboratory personnel. Most importantly, it helps reduce unnecessary loss of blood supply due to outdating of blood.

An MSBOS is a list of the maximum number of RBC units that the blood bank will crossmatch usually preoperatively for a specific procedure, as well as procedures for which a T and S is appropriate.[5] Implementation of MSBOS helps in the appropriate and logical use of blood bank supplies, avoids misplacement of blood requests for surgeries where preoperative blood requirement is minimal, and helps ensure availability of adequate blood in emergency situations. It also eliminates the need to determine the number of blood components for each case, reduces too late blood requests that do not allow sufficient time for component preparation, decreases pretransfusion compatibility testing, and avoids expiry of components.

Blood is a precious and limited commodity, dependent on finite public donations and requires to be used effectively to avoid misuse and wastage. The hospital blood banks and the hospital transfusion committees in particular have a unique role in the initiation and formulation of blood ordering schedules. A strong institutional commitment is required to lift the profile of blood transfusion. The implementation of such schedules requires a careful assessment of blood utilization practices of the hospital in the preceding 6 months. Once implemented, review at regular intervals of 3 or 6 months is crucial to assess the impact of the newly executed policies. Discrepancies at any level must be carefully reassessed and modification in the program (if any) must be promptly instituted to prevent additional loss of blood supplies.

MSBOS is subjected to constant improvement and updated with inputs from all quarters, namely, the treating clinicians, anesthetists, surgeons, nurses, as well as the blood bank staff with an overall guidance from the hospital transfusion committee. These efforts and contributions are the key for better transfusion practices in the hospital. Apart from the constant surveillance from the hospital transfusion committee, hospital educational programs may be developed for improvement of blood transfusion practices and to provide a framework for blood bank audit. These educational programs include circulation of information sheets, reminders, regular audit, as well as adoption of revised guidelines.

The system of SBOS allows for flexibility in the sense that should an antibody screen results be positive, antigen-negative, crossmatched blood must be made readily available. These blood ordering schedules are subject to approval by both the hospital transfusion committee and the clinicians. The efficiency of any blood ordering schedule implementation would require a similar audit after a minimum period of 6 months. The present schedule was presented before the hospital transfusion committee and approval was obtained from both the committee members and Orthopedicians.

A sufficient number of patients were not available for surgeries, such as tension-band wiring and lumbar discectomy (n < 5), which may have biased our final result in making the blood ordering schedule. However, as an initial measure it would prove to cut down unnecessary costs of reagents, resources, and the workload of blood bank personnel. Prospective studies would be required to find the efficiency of such SBOEs in other elective procedures. Also, for purposes of ease and simplicity we chose to employ the SBOE in the present study. Other factors, such as low weight, short stature, which may predict the risk of allogenic transfusion, were not taken in to account in this SBOE.

## CONCLUSIONS

We had 41% of crossmatched blood being utilized for elective orthopedic surgery. A high C:T (>2.5) ratio was observed in internal fixation with compression plating, dynamic compression screw, dynamic hip screw, lumbar discectomy and hip prosthetic replacement. The calculated RBC units were less than 0.5 units in 5 of the 12 elective procedures and hence we recommend a group and save policy for them. In order to reduce unnecessary crossmatching, blood ordering schedule catering to surgeon and patient requirements is the need of the hour. It is essential for every institutional blood bank to formulate a blood ordering schedule in conjunction with the clinicians for appropriate blood usage. Regular auditing and periodic feedbacks are also essential to improve the blood utilization practices.

## References

[1] Bhutia SG, Srinivasan K, Ananthakrishnan N, Jayanthi S, Ravishankar M (1997). Blood utilization in elective surgery–requirements, ordering and transfusion practices. Natl Med J India.

[2] Chawla T, Kakepoto GN, Khan MA (2001). An audit of blood cross-match ordering practices at the Aga Khan University Hospital: first step towards a Maximum Surgical Blood Ordering Schedule. J Pak Med Assoc.

[3] Vibhute M, Kamath SK, Shetty A (2000). Blood utilization in elective general surgery cases: Requirements, ordering and transfusion practices. J Postgrad Med.

[4] Friedman BA, Oberdman HA, Chandnick AR, Kingdon KI (1976). The maximum surgical blood order schedule and surgical blood use in United States. Transfusion.

[5] Friedman BA (1979). An analysis of Surgical Blood use in United States hospitals with application to the maximum surgical blood order schedule. Transfusion.

[6] Guidelines for implementation of a maximum surgical blood order system schedule (1990). The British Committee for Standards in Haematology Blood Transfusion Task Force. Clin Lab Haematol.

[7] Murphy WG, Phillips P, Gray A, Heatley L, Palmer J, Hopkins D (1995). Blood use for surgical patients: a study of Scottish hospital transfusion practices. J R Coll Surg Edinb.

[8] Bierbaum BE, Callaghan JJ, Galante JO, Rubash HE, Tooms RE, and Welch RB (1999). An analysis of blood management in patients having a total hip or knee arthroplasty. J Bone Joint Surg Am.

[9] (1994). Use of blood products for elective surgery in 43 European hospitals. The Sanguis Study Group. Transfus Med.

[10] Nuttall GA, Santrach PJ, Oliver WC, Ereth MH, Horlocker TT, Cabanela ME (2000). Possible guidelines for autologous red blood cell donations before total hip arthroplasty based on the surgical blood order equation. Mayo Clin Poc.

[11] Huber-Wagner S, Qvick M, Mussack T, Euler E, Kay MV, Mutschler W (2007). Massive blood transfusion and outcome in 1062 polytrauma patients a prospective study based on the trauma registry of the German Trauma Society. Vox Sang.

[12] Mead JH, Anthony CD, Sattler M (1980). Hemotherapy in elective surgery: an incidence report, review of literature and alternatives for guideline appraisal. Am J Clin Path.

[13] Knowles S (2007). Blood transfusion : challenges and limitations. Transfus Altern Transfus Med.

[14] Nuttall GA, Horlocker TT, Santrach PJ, Oliver WC, Dekutoski MB, Bryant S (2000). Use of the surgical blood order equation in spinal instrumentation and fusion surgery Spine.

[15] Sakurai Y, Okada C (2001). Comparison by simulation of the efficiency of surgical blood order equation with that of maximum surgical blood order schedule. Masui.

[16] Gupta PK, Kumar H, Diwan RN (2003). Blood ordering strategies in the Armed forces: a proposal. Med J Armed Forces India.

[17] Wong L, Cheng G (1995). Type and screen of blood units at a teaching hospital. Hong Kong Med J.

[18] Boral LI, Henry JB (1977). The type and screen: a safe alternative and supplement in selected surgical procedures. Transfusion.

[19] Petrides M, Stack G (2001). Practical guide to transfusion medicine.

